# Activation of Inflammatory and Apoptosis Pathways on Human Gingival Fibroblasts Exposed to Dental Resin Composites

**DOI:** 10.3390/polym17202779

**Published:** 2025-10-17

**Authors:** Francesco De Angelis, Edoardo Sorrentino, Antonella Mazzone, Ylenia Della Rocca, Jacopo Pizzicannella, Oriana Trubiani, Giovanna Iezzi, Camillo D’Arcangelo, Guya Diletta Marconi, Francesca Diomede

**Affiliations:** 1 Department of Medical, Oral and Biotechnological Sciences, University “G. d’Annunzio” Chieti-Pescara, 66100 Chieti, Italy; giovanna.iezzi@unich.it (G.I.); camillo.darcangelo@unich.it (C.D.); 2 Department of Innovative Technologies in Medicine and Dentistry, University “G. d’Annunzio” Chieti-Pescara, 66100 Chieti, Italy; edoardo.sorrentino@unich.it (E.S.); antonella.mazzone@unich.it (A.M.); ylenia.dellarocca@unich.it (Y.D.R.); jacopo.pizzicannella@unich.it (J.P.); oriana.trubiani@unich.it (O.T.); guya.marconi@unich.it (G.D.M.); francesca.diomede@unich.it (F.D.)

**Keywords:** dental resins, human gingival fibroblasts, inflammation, apoptosis

## Abstract

The use of dental composite resins has significantly increased over recent years, thanks to their esthetics and mechanical features, despite some doubts being raised about their biocompatibility. Residual methacrylate can be eluted from bulk composites, and its amount may significantly increase over time, leading to cytotoxic effects that can involve several inflammatory patterns. The aim of this in vitro study was to evaluate the activation of the inflammatory pathway NFκB p65/MyD88/NALP3 and the apoptosis pathway of BCL-2/BAX/Caspase-3 (CASP-3) expression on human gingival fibroblasts (hGFs) after 24 h and 1-week exposure to the eluates of three heat-cured dental composite resins: GrandioSO, VOCO (GR); Enamel Plus HRi Biofunction, Micerium (BF); and Filtek universal restorative, 3M (FU). The results highlighted that NFκB p65/MyD88/NALP3 was activated after resin exposure in a time-dependent manner. Moreover, immunofluorescence and gene expression analyses showed that pro-apoptotic markers CASP-3 and BAX were elevated, while anti-apoptotic protein BCL-2 was suppressed in hGFs after dental resin exposure. The present in vitro study analyzed mechanisms related to cytotoxicity and apoptosis, suggesting ways to limit composite cytotoxicity through advancements in material technology. The activation of inflammation and apoptotic pathways appeared to be material-dependent, and was less pronounced with BF and FU, which could hypothetically indicate them being a safer clinical choice to preserve periodontal health in daily dental practice.

## 1. Introduction

Biocompatibility is defined as a material’s ability to perform its medical function, leading to no clinically significant adverse effects and promoting an adequate cellular or tissue response [1]. A dental material considered as “ideal” should not be cytotoxic. Nowadays, the use of dental composite resins has significantly increased due to their enhanced mechanical properties and to a higher demand for minimally invasive and esthetic restorations. However, their application has raised some doubts about biocompatibility [2,3,4,5,6].

Resin-based dental composites are made up of an organic matrix, fillers, pigments, silanic agent, catalysts, and inhibitors [7]. Many organic matrix monomers, such as bisphenol-A-glycidyl-methacrylate (Bis-GMA), triethylene glycol dimethacrylate (TEGDMA), 2-hydroxyethyl methacrylate (HEMA), and urethane dimethacrylate (UDMA), have been investigated for their ability to affect the ultimate biocompatibility of the material [6,8,9,10]. Bis-GMA is one of the most-employed monomers, due to its many advantageous properties, including flexural strength, water sorption/solubility, volumetric shrinkage, and viscosity [11]. Nevertheless, Bis-GMA and other bisphenol A (BPA)-derived monomers can promote cytotoxicity and genotoxicity through cytokine release/inhibition and DNA damage, ultimately inducing necrosis and apoptosis [12,13].

Cytotoxicity is often related to a pro-inflammatory reaction after PGE2 production and COX2 expression, increasing the release of pro-inflammatory cytokines [14,15,16]. The inflammatory pathway NFκB p65/MyD88/NALP3 involves the transcription factor of the nuclear factor kappa-light-chain-enhancer of activated B cells (NFκB); the inflammasome protein NOD-, LRR-, and pyrin domain-containing 3 (NALP3); and the pro-inflammatory cytokine interleukin-1 beta (IL-1β). This pathway covers different sides of the innate and adaptive immune system and inflammation [17,18]. NFκB modulates NALP3 inflammasome activation, working as a factor of transcription promoting NALP3 expression [19]. Inflammasome is activated by its components’ overexpression, which can be promoted by pathogen-associated molecular patterns (PAMPs) or damage-associated molecular patterns (DAMPs), through pattern recognition receptors (PRRs) such as TLRs.

Cytotoxicity caused by DNA integrity violation is closely related to apoptosis [20]. Apoptosis takes place over development and aging, as a homeostatic process, and acts as a defense mechanism in immune responses or cellular injury due to disease or harmful agents [21]. Apoptosis pathways are regulated by different factors, especially those concerning the B cell lymphoma 2 (BCL-2), BAX, and Caspase-3 (CASP-3) [22]. The pro-apoptotic BAX factor regulates the intrinsic apoptosis pathway caused by the induction of cytochrome C release from mitochondria and cell death [23]. BCL-2 proteins regulate and perform the intrinsic or mitochondrial apoptosis pathways, and also affect the extrinsic pathway and necrotic cell death [24]. CASP-3 is an activated death protease that catalyzes the cleavage of many key cellular proteins [25], specifically promoting stress-induced tumor cell proliferation, cell migration, invasive potential, and cancer angiogenesis [26,27,28,29]. CASP-3 promotes DNA strand break and fragmentation [20,30,31] and may be triggered via intrinsic or extrinsic pathways. Specifically, the intrinsic pathway is related to Caspase-9 activation, promoted by mitochondria disruption, while the extrinsic pathway is modulated by Caspase-8 through activation of the death receptor [20]. Cleaved CASP-3 are apoptotic pathway executors, and their activation is essential for the induction of apoptosis [22].

Understanding and highlighting the cytotoxic risk behind the use of materials that are extensively employed in daily dental practice, such as composite resins, seems to be of primary relevance, especially from the clinical point of view. Dental restorative materials are thought to stay within the oral environment for a long time, and if they are not sufficiently biocompatible, they may easily lead to toxic or allergic reactions [32,33,34]. Oral health could hypothetically be impaired due to a direct contact between composite restorations and the periodontium, leading to gingival inflammation and recession [35], or due to monomer elution and diffusion, also through the dentinal tubules, with consequent pulp reactions that may ultimately compromise the vitality of the pulp [36]. Many studies have demonstrated an inflammation onset due to the presence of restoration close to the periodontium [35,37,38,39,40]. With the purpose of promoting the development and the selection of products able to minimize or even eliminate the triggering of inflammatory and apoptotic pathways on exposed cells, the aim of this in vitro study was to examine the activation of the NFκB p65/MyD88/NALP3 pathway and the BCL-2/BAX/CASP-3 apoptosis pathway expression on human gingival fibroblasts (hGFs) after 24 h and 1-week exposure to the eluates of three commercial dental composite resins. The null hypothesis tested was that no differences in terms of pathway activation could be detected when comparing the three composite resin eluates to one another or when comparing them to the control group, which included hGFs not exposed to any composite eluate.

## 2. Materials and Methods

An overview of the composite resins selected for each different experimental group and their corresponding formulations is given in Table 1.

### 2.1. Sample Preparation for Eluates Deriving from GR, BF, and FU

Cylindrical composite disks (diameter: 4 mm, height: 2 mm) with a total surface area of 50.27 mm^2^ were manufactured for each material by placing the uncured composite material into polyvinylsiloxane molds. Different molds were employed for each different material, to avoid contamination. Two microscope slides were positioned over and below the silicone molds and clamped with a paper clip for 20 s to squeeze out the over-material. A light curing unit (Celalux 3, VOCO, Cuxhaven, Germany) with an 8 mm tip diameter and 1300 mW/cm^2^ output power was employed to light-cure specimens for 20 s on the upper surface. After cleaning each disk with an ultrasonic bath, specimens were exposed to a further heat-curing cycle in a composite heat-curing unit at 80 °C for 10 min (LaborLux, Micerium, Avegno, Genova, Italy).

Fifty samples of each group were added to a 50 mL falcon containing 125 mL of complete fibroblast basal medium and left at 37 °C and 100% humidity for 1 week. Then, the eluates were removed from the resins and left at 4 °C, awaiting future use. The eluate was then mixed with the culture medium in a ratio of 1:1 and subsequently added to the plates containing the hGFs [41].

### 2.2. Cell Culture Establishment

Human gingival fibroblasts (hGFs) primary cultures were established by the explant procedure. Healthy gingival tissue fragments were rinsed three times in Phosphate-Buffered Saline (PBS, Lonza, Basel, Switzerland) solution, cut into pieces and cultured in Dulbecco’s modified Eagle’s medium (DMEM, Lonza) supplemented with 10% Fetal Bovine Serum (FBS, Lonza) and 0.1% gentamicin (10 mg/mL; Euroclone, Milan, Italy) at 37 °C in a 5% CO_2_ atmosphere. Gingival biopsies were cultured until the spontaneous migration of hGFs (about 4 weeks), as previously reported [42]. Cells were incubated in standard conditions (37 °C in a humidified atmosphere of 5% (*v*/*v*) CO_2_). All the experiments were performed with cells processed between four and eight passages, and each assay was performed in triplicate.

### 2.3. Cell Metabolic Activity

The 3-(4,5-dimethylthiazol-2-yl)-5-(3-carboxymethoxyphenyl)-2-(4-sulfo-phenyl)-2H-tetrazolium (MTS) assay (CellTiter 96^®^ Aqueous One Solution Cell Proliferation Assay, Promega, Madison, WI, USA) was employed to investigate the cell metabolic activity of hGFs cultured alone (Control Group, CTRL) and/or with GR, BF, and FU resin eluates. For the MTS assay, 2.4 × 10^3^ hGFs/well were seeded into 96-well plates and maintained in culture at 37 °C with DMEM (Lonza) supplemented with 10% of FBS (Lonza) for 24 h, 48 h, 72 h, and 1 week. After these respective culture times, 20 μL/well of MTS staining solution was added and the plates were incubated at 37 °C for 3 h. Quantification of formazan salts, directly related to cell viability, was measured through absorbance at a wavelength of 490 nm using the Synergy™ HT Multi-detection microplate reader (Biotech, Winooski, VT, USA). The MTS assay was performed in triplicate [43].

### 2.4. Experimental Design

For all the subsequent experiments, hGFs were cultured with and without the eluates derived from GR, BF, and FU for 24 h and 1 week. Each experimental point was performed in triplicate on:

- hGFs cultured with complete medium for 24 h (Control Groups, CTRL);
- hGFs cultured with eluate derived from GR for 24 h;
- hGFs cultured with eluate derived from BF for 24 h;
- hGFs cultured with eluate derived from FU for 24 h;
- hGFs cultured with complete medium for 1 week;
- hGFs cultured with eluate derived from GR for 1 week;
- hGFs cultured with eluate derived from BF for 1 week;
- hGFs cultured with eluate derived from FU for 1 week.

### 2.5. Immunofluorescence Staining for Confocal Laser Scanning Microscope (CLSM) Detection

hGFs cultured without (CTRL) and with eluates were seeded into an 8-well culture glass slide (Corning, Glendale, AZ, USA) at a density of 6.4 × 10^4^/well. After 24 h and 1 week, the cells were fixed with 0.4% paraformaldehyde (PFA) (BioOptica, Milan, Italy) for 45 min at room temperature. As soon as the samples were washed three times in PBS, they were permeabilized for 6 min in 0.1% Triton X-100 (BioOptica), blocked for 1 h with 5% of non-fat milk in PBS and incubated overnight at 4 °C with the primary antibodies anti-NFκB p65 (sc-8008, Santa Cruz Biotechnology, Dallas, TX, USA), anti-MyD88 (sc-136970, Santa Cruz Biotechnology), anti-NALP3 (sc-134306, Santa Cruz Biotechnology), anti-BCL-2 (sc-7382, Santa Cruz Biotechnology), anti-BAX (sc-493, Santa Cruz Biotechnology), and anti-CASP-3 (sc-56052, Santa Cruz Biotechnology) at a concentration of 1:200. Subsequently, all the samples were first incubated with the secondary antibodies Goat anti-Mouse IgG (H + L) Highly Cross-Adsorbed Secondary Antibody, Alexa Fluor 568 (A11031, Invitrogen, Thermo Fisher Scientific, Waltham, MA, USA), and Goat anti-Rabbit IgG (H + L) Cross-Adsorbed Secondary Antibody, Alexa Fluor 568 (A11011, Invitrogen) at a concentration of 1:200 1 h at 37 °C, then with Alexa Fluor 488 phalloidin green fluorescent conjugate (A12379, Invitrogen) and TOPRO (T3605, Invitrogen) to stain, respectively, actin and nuclei. The images were captured through the Zeiss LSM800 confocal system (Carl Zeiss, Jena, Germany) equipped with a Plan Neofluar oil-immersion objective (40 × 1.3 NA). Images were collected using an argon laser beam with excitation lines at 488 nm and a helium–neon source (543 and 665 nm) with an image size resolution of 1024 × 1024 pixels [42].

### 2.6. RNA Isolation and Real-Time RT-PCR

PureLink RNA Mini Kit (Ambion, Thermo Fisher Scientific, Milan, Italy) were employed to extract total RNA of all the samples. Once RNA was obtained, the retro transcription of each sample was performed using M-MLV Reverse Transcriptase (M1302 Sigma-Aldrich, Saint Louis, MO, USA). Specifically, 1 µg of total RNA was used to obtain 50 µg of cDNA, as recommended in the technical bulletin. Then, the Mastercycler ep real plex Real-Time PCR system (Eppendorf, Hamburg, Germany) was employed to execute a Real-Time PCR. The cDNA from the samples were amplificated following the steps below: 3 min 95 °C, 40 cycles of 15 s at 95 °C, and 1 min at 60 °C to evaluate gene expression of *RELA* (Hs.PT.58.22880470 IDT), *MYD88* (Hs.PT.58.40428647.g), *NLRP3* (Hs.PT.58.39303321), *BCL2* (Hs.PT.56a.2905156), *BAX* (Hs.PT.56a.19141193), and *CASP3* (Hs.PT.56a.25882379.g). *B2M* (Hs.PT.58v.18759587) expression was evaluated in all the samples as the housekeeping gene. All the primers were purchased from Integrated DNA Technologies (IDT, Tema Ricerca, Bologna, Italy) (Table 2). The expression levels for each gene were obtained through the 2^−ΔΔCt^ method. Real-Time PCR was performed in three independent experiments [44].

### 2.7. Statistical Analysis

The statistical analysis was carried out using one-way ANOVA and post hoc Tukey’s multiple comparisons test using GraphPad 5 (GraphPad, San Diego, CA, USA) software. All differences were considered statistically significant for values of *p* < 0.05. The comparative 2^−ΔΔCt^ method was employed to obtain gene expression data after real-time PCR.

## 3. Results

### 3.1. Effects of Dental Composites on hGFs

**Figure 1:**
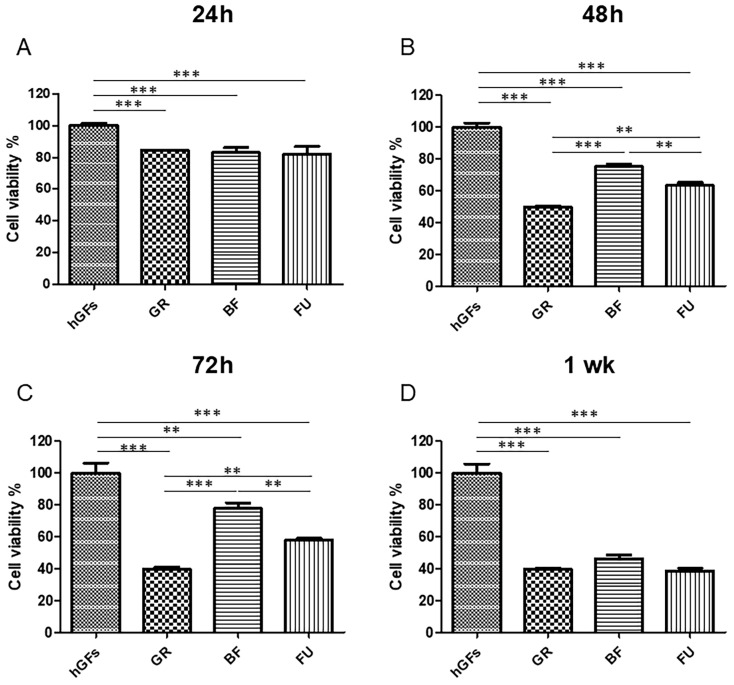
Metabolic activity of the cells. Effect of GR, BF, and FU on hGFs after 24 h, 48 h, 72 h, and 1 week of treatment. (**A**) Cell viability analysed at 24h of culture. (**B**) Cell viability analysed at 48h of culture. (**C**) Cell viability analysed at 72h of culture. (**D**) Cell viability analysed after 1 week of culture. Results are shown as mean ± standard error (SEM) (*n* ≥ 3) of the optical percentage (%) of metabolically active cells (** *p* < 0.01, *** *p* < 0.001).

The hGFs exposed to GR, BF, and FU eluates for 24 h, 48 h, 72 h, and 1 week showed a general trend towards the metabolic activity reduction. After 24 h and 48 h exposure, the eluates induced just a slight decrease in metabolic activity, while after 72 h and 1 week, the metabolic activity reduction was statistically significant when compared to the basal condition (CTRL) (Figure 1).

### 3.2. Dental Composite Treatment Modulated the Pro-Inflammatory and Apoptotic Markers in hGFs Culture

**Figure 2:**
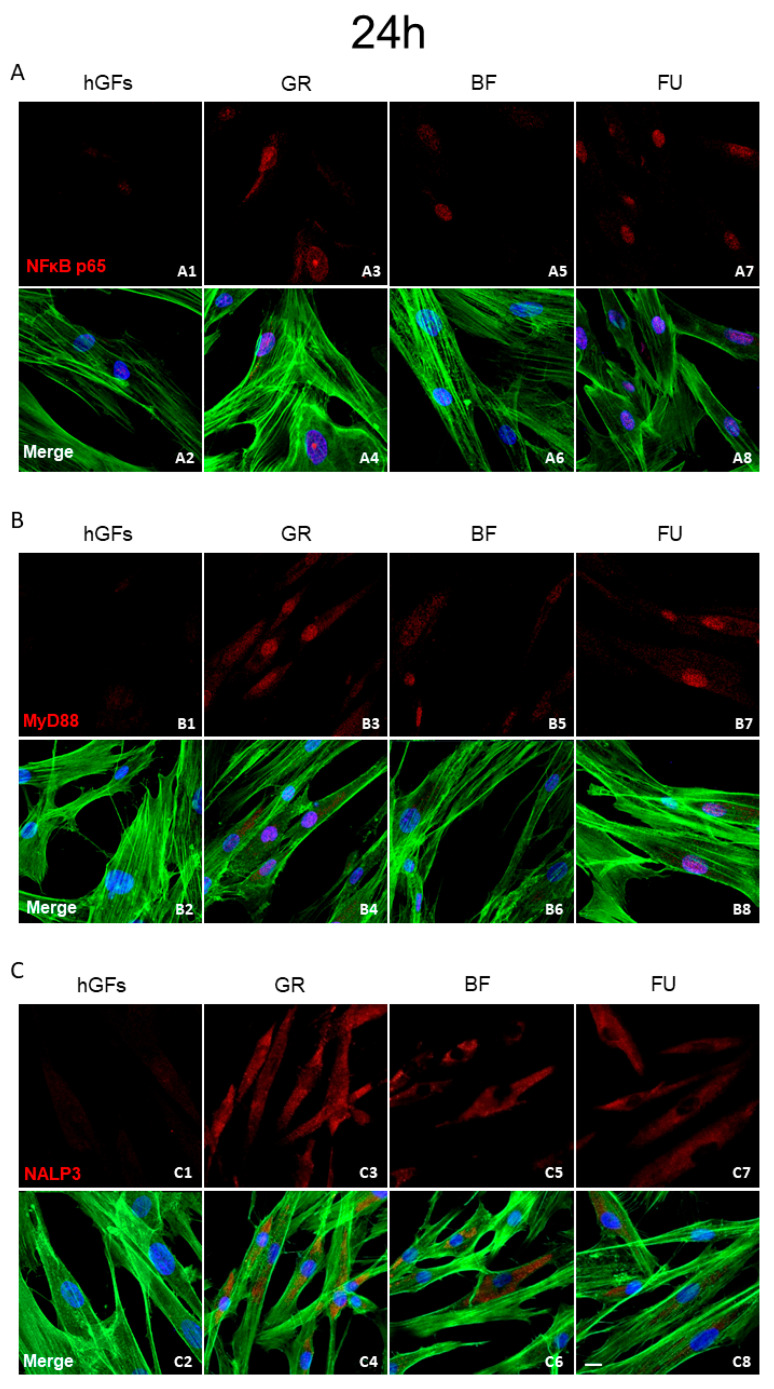
Expression of inflammatory markers after 24 h of treatment. (**A**) Immunofluorescence detection of NFkB, red channel, in the CTRL group (**A1**) and after 24 h of exposure to GR (**A3**), BF (**A5**), and FU (**A7**); merge image of all channels in the CTRL group (**A2**) and after 24 h of exposure to GR (**A4**), BF (**A6**), and FU (**A8**). (**B**) Immunofluorescence detection of MyD88, red channel, in the CTRL group (**B1**) and after 24 h of exposure to GR (**B3**), BF (**B5**), and FU (**B7**); merge image of all channels in the CTRL group (**B2**) and after 24 h of exposure to GR (**B4**), BF (**B6**), and FU (**B8**). (**C**) Immunofluorescence detection of NALP3, red channel, in the CTRL group (**C1**) and after 24 h of exposure to GR (**C3**), BF (**C5**), and FU (**C7**); merge image of all channels in the CTRL group (**C2**) and after 24 h of exposure to GR (**C4**), BF (**C6**), and FU (**C8**). Mag: 40×. Scale bar: 10 µm.

**Figure 3:**
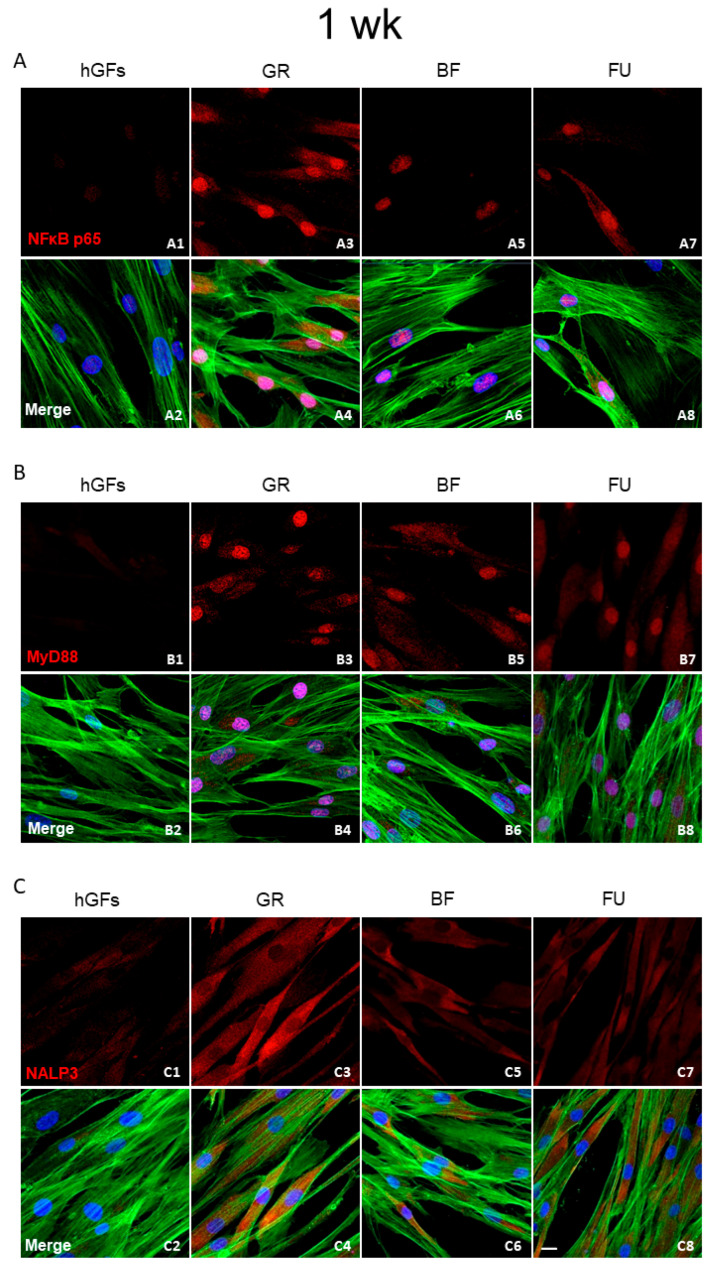
Expression of inflammatory markers after 1 week of treatment. (**A**) Immunofluorescence detection of NFkB, red channel, in the CTRL group (**A1**) and after 1 week of exposure to GR (**A3**), BF (**A5**), and FU (**A7**); merge image of all channels in the CTRL group (**A2**) and after 1 week of exposure to GR (**A4**), BF (**A6**), and FU (**A8**). (**B**) Immunofluorescence detection of MyD88, red channel, in the CTRL group (**B1**) and after 1 week of exposure to GR (**B3**), BF (**B5**), and FU (**B7**); merge image of all channels in the CTRL group (**B2**) and after 1 week of exposure to GR (**B4**), BF (**B6**), and FU (**B8**). (**C**) Immunofluorescence detection of NALP3, red channel, in the CTRL group (**C1**) and after 1 week of exposure to GR (**C3**), BF (**C5**), and FU (**C7**); merge image of all channels in the CTRL group (**C2**) and after 1 week of exposure to GR (**C4**), BF (**C6**), and FU (**C8**). Mag: 40×. Scale bar: 10 µm.

**Figure 4:**
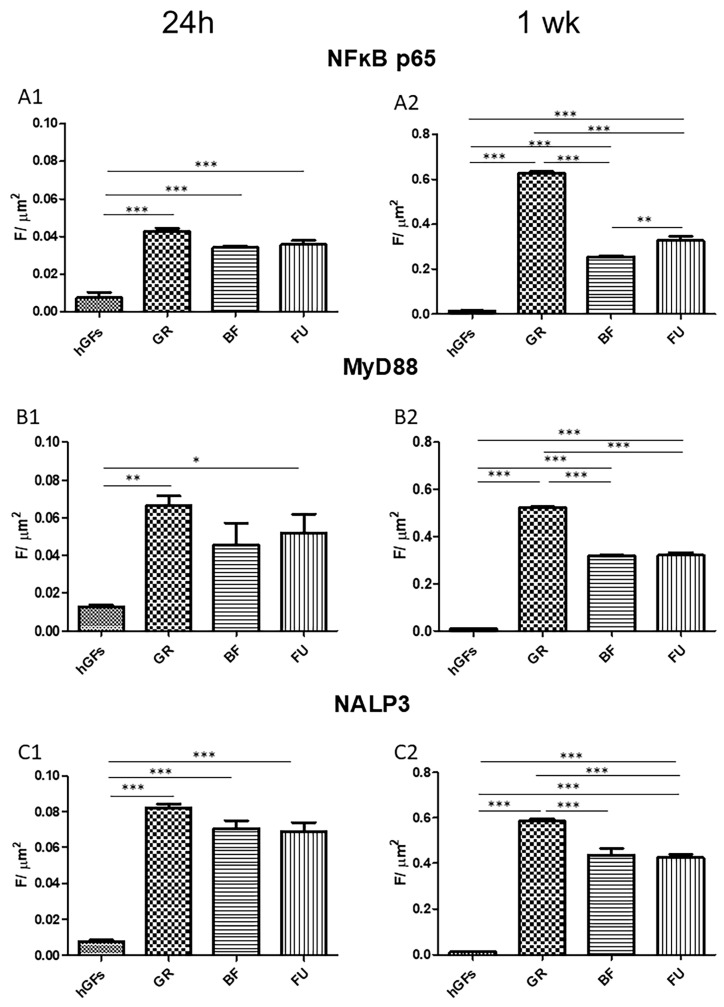
Quantization of expression of inflammatory markers after 24 h and 1 week of treatment. Quantitative analyses of NFkB p65 expression after 24 h (**A1**) and 1 week (**A2**); MyD88 expression after 24 h (**B1**) and 1 week (**B2**); NALP3 expression after 24 h (**C1**) and 1 week (**C2**). Immunofluorescence quantitative analysis expression was calculated as arbitrary unit of fluorescence per cell surface unit (F/μm^2^). Data are expressed as mean ± S.E.M. * *p* < 0.05; ** *p* < 0.01; *** *p* < 0.001.

The immunofluorescence revealed that in the hGFs culture, the pro-inflammatory markers levels NFkB p65, MyD88, and NALP3 were upregulated after exposure to GR, BF, and FU eluates (Figure 2, Figure 3 and Figure 4). In particular, the level of inflammatory proteins was significantly upregulated after 1-week exposure. The inflammation pathway was more upregulated following the GR eluate exposure compared to the BF and FU eluates (Figure 3 and Figure 4).

**Figure 5:**
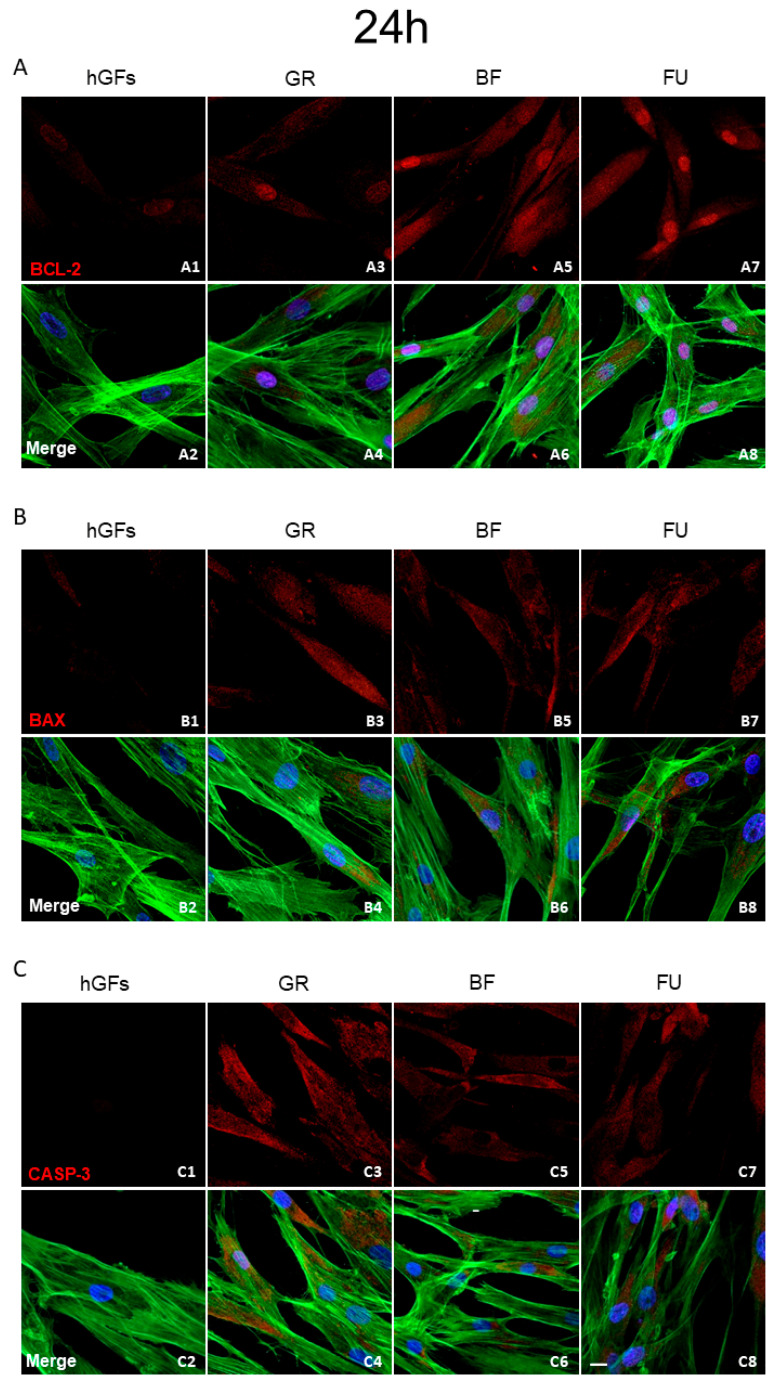
Expression of apoptotic markers after 24 h of treatment. (**A**) Immunofluorescence detection of BCL-2, red channel, in the CTRL group (**A1**) and after 24 h of exposure to GR (**A3**), BF (**A5**), and FU (**A7**); merge image of all channels in the CTRL group (**A2**) and after 24 h of exposure to GR (**A4**), BF (**A6**), and FU (**A8**). (**B**) Immunofluorescence detection of BAX, red channel, in the CTRL group (**B1**) and after 24 h of exposure to GR (**B3**), BF (**B5**), and FU (**B7**); merge image of all channels in the CTRL group (**B2**) and after 24 h of exposure to GR (**B4**), BF (**B6**), and FU (**B8**). (**C**) Immunofluorescence detection of CASP-3, red channel, in the CTRL group (**C1**) and after 24 h of exposure to GR (**C3**), BF (**C5**), and FU (**C7**); merge image of all channels in the CTRL group (**C2**) and after 24 h of exposure to GR (**C4**), BF (**C6**), and FU (**C8**). Mag: 40×. Scale bar: 10 µm.

**Figure 6:**
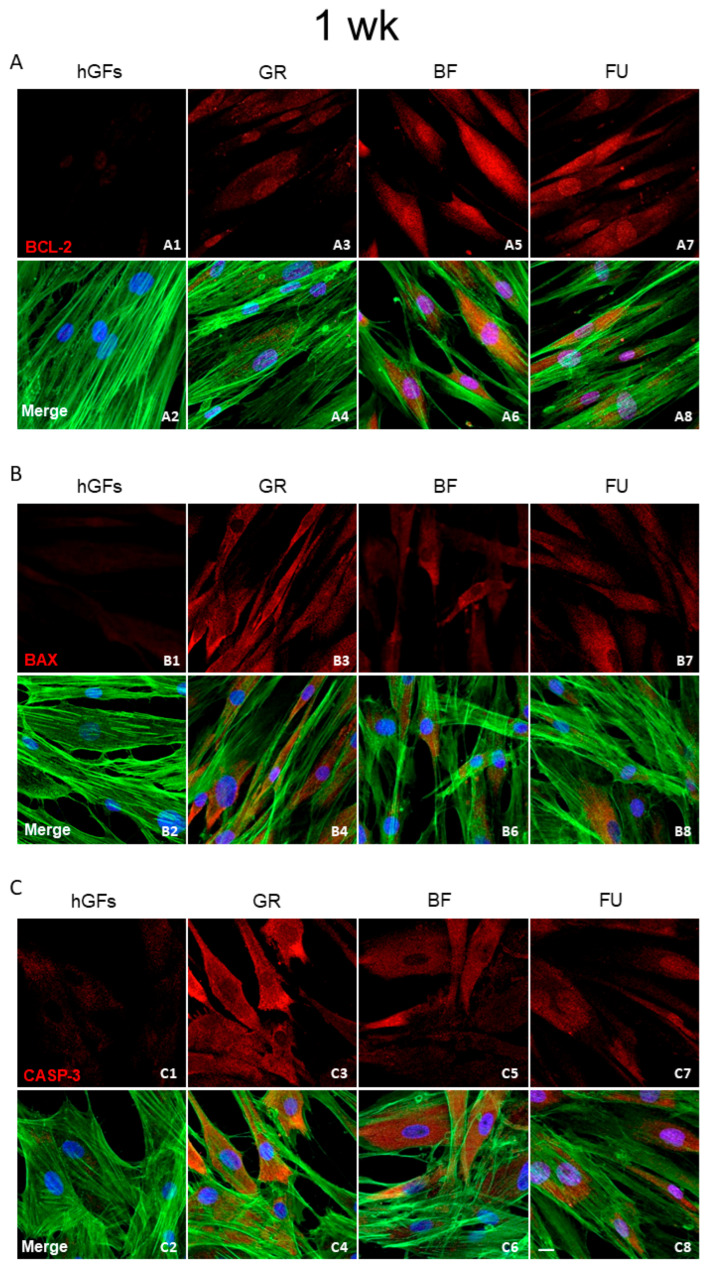
Expression of apoptotic markers after 1 week of treatment. (**A**) Immunofluorescence detection of BCL-2, red channel, in the CTRL group (**A1**) and after 1 week of exposure to GR (**A3**), BF (**A5**), and FU (**A7**); merge image of all channels in the CTRL group (**A2**) and after 1 week of exposure to GR (**A4**), BF (**A6**), and FU (**A8**). (**B**) Immunofluorescence detection of BAX, red channel, in the CTRL group (**B1**) and after 1 week of exposure to GR (**B3**), BF (**B5**), and FU (**B7**); merge image of all channels in the CTRL group (**B2**) and after 1 week of exposure to GR (**B4**), BF (**B6**), and FU (**B8**). (**C**) Immunofluorescence detection of CASP-3, red channel, in the CTRL group (**C1**) and after 1 week of exposure to GR (**C3**), BF (**C5**), and FU (**C7**); merge image of all channels in the CTRL group (**C2**) and after 1 week of exposure to GR (**C4**), BF (**C6**), and FU (**C8**). Mag: 40×. Scale bar: 10 µm.

**Figure 7:**
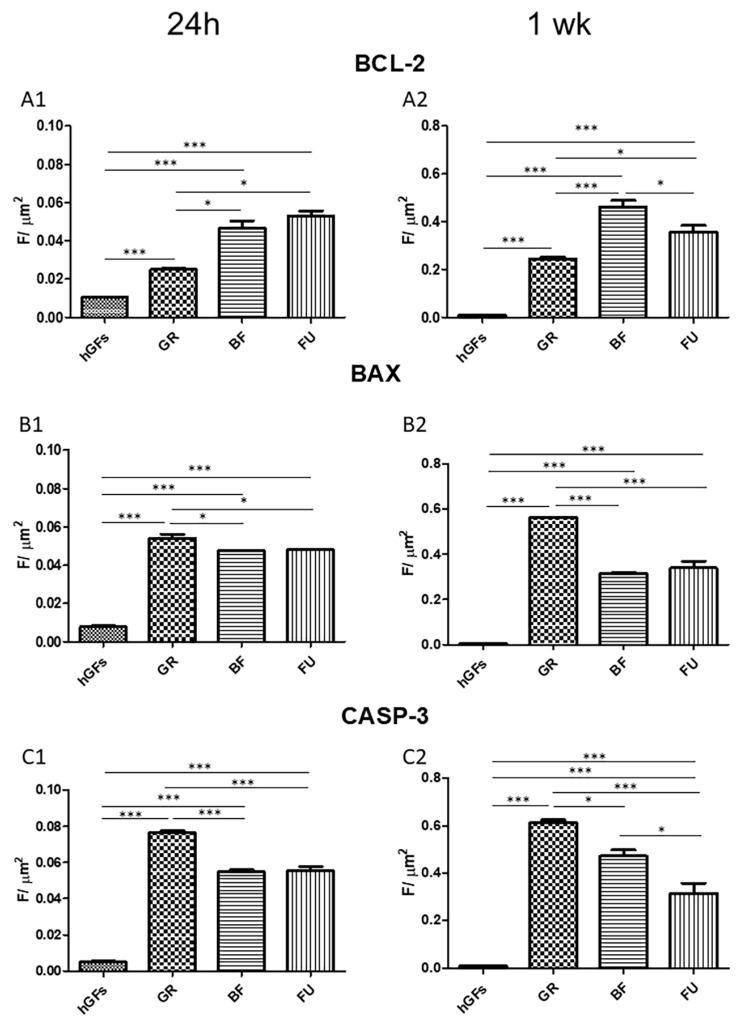
Quantization of expression of apoptotic markers after 24 h and 1 week of treatment. Quantitative analyses of BCL-2 expression after 24 h (**A1**) and 1 week (**A2**); BAX expression after 24 h (**B1**) and 1 week (**B2**); CASP-3 expression after 24 h (**C1**) and 1 week (**C2**). Immunofluorescence quantitative analysis expression was calculated as arbitrary unit of fluorescence per cell surface unit (F/μm^2^). Data are expressed as mean ± S.E.M. * *p* < 0.05; *** *p* < 0.001.

The level of BCL-2 immunofluorescence was decreased in hGFs exposed to all composite eluates both for 24 h and 1 week (Figure 5A and Figure 6A). Specifically, BCL-2 expression decreased in hGFs exposed to GR when compared to untreated cells (CTRL) and to BF and FU exposure (Figure 7A1,A2). On the other hand, the levels of BAX and CASP-3 increased after dental composites exposure (GR, BF, and FU) (Figure 5B,C and Figure 6B,C). Quantitative data for BAX and CASP-3 expression showed increased levels after 1-week exposure to BF and FU eluates (Figure 7B1,B2,C1,C2).

**Figure 8:**
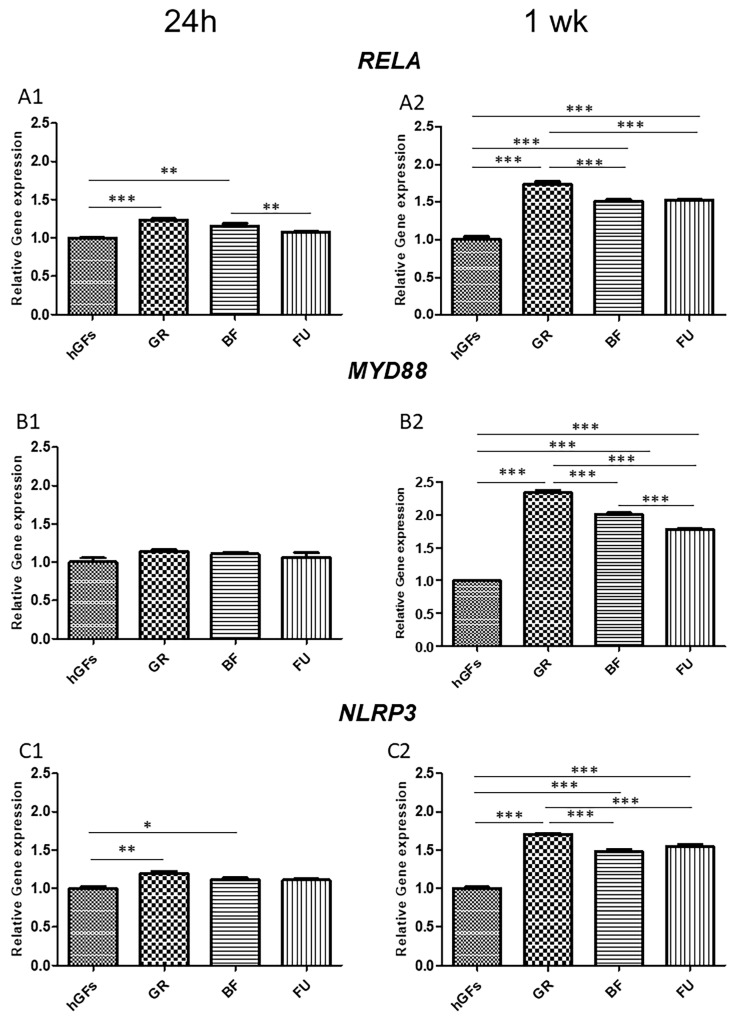
*RELA*, *MYD88*, and *NLRP3* gene expression. (**A1**,**B1**,**C1**) Histograms of real-time PCR for *RELA-*, *MYD88-*, and *NLRP3-*related gene expression in untreated cells (CTRL) and GR, BF, and FU samples after 24 h of exposure. (**A2**,**B2**,**C2**) Histograms of real-time PCR for *RELA-*, *MYD88-*, and *NLRP3*-related gene expression in untreated cells (CTRL) and GR, BF, and FU samples after 1 week of exposure. * *p* < 0.05; ** *p* < 0.01; *** *p* < 0.001.

**Figure 9:**
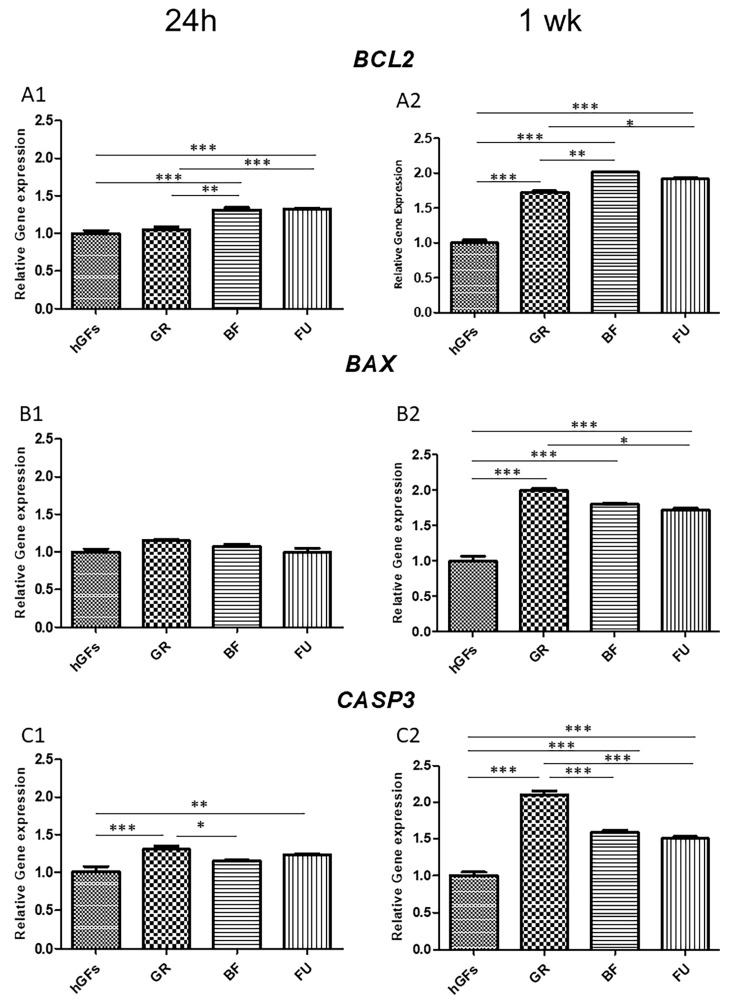
*BCL2*, *BAX,* and *CASP3* gene expression. (**A1**,**B1**,**C1**) Histograms of real-time PCR for *BCL2*-, *BAX*- and *CASP3*-related gene expression in untreated cells (CTRL) and GR, BF, and FU samples after 24 h of exposure. (**A2**,**B2**,**C2**) Histograms of real-time PCR *BCL2*-, *BAX*- and *CASP3*-related gene expression in untreated cells (CTRL), and GR, BF, and FU samples after 1 week of exposure. * *p* < 0.05; ** *p* < 0.01; *** *p* < 0.001.

The data from RT-PCR analysis on *RELA*, *MYD88*, *NLRP3*, *BCL2*, *BAX,* and *CASP3* expression supported the results from Confocal Microscopy (Figure 8 and Figure 9). In addition, the real-time analysis highlighted that, after 1 week of exposure to GR eluate, the *RELA*, *MYD88*, and *NLRP3* levels of expression were upregulated when compared to untreated cells (CTRL) and to BF- and FU-exposed hGFs (Figure 8A2,B2,C2).

On hGFs exposed for 1 week to GR eluates, the gene expression analysis of *BAX* and *CASP3* highlighted a significant pro-apoptotic upregulation (Figure 9B2,C2). On the other hand, after 1 week of exposure to GR eluates, the gene expression of *BCL2* was downregulated when compared to all the other groups (Figure 9A2).

## 4. Discussion

This in vitro study focused on the cytotoxic effects promoted on hGF cells by the eluates of three commercially available dental resin composites (GR, BF, and FU) after 24 h/1-week exposure. The null hypotheses tested had to be rejected. Differences in terms of pathway activation were detected when comparing the composite eluates to one another and when comparing them to the control group. Previous studies have already analyzed the potential cytotoxicity of dental composite resins, but many of them focused just on the specific effect of each individual material component [45,46,47]. Composite eluates, on the other hand, allow us to study the effect of all of the several components in dilution. Compared to single-component experiments, the eluates model can mimic more closely the cell exposition that takes place in the oral cavity ecosystem [8]. Moreover, any interactive effect existing among the different components and affecting the overall material toxicity may also be investigated [8].

As established with MTS analysis, hGFs exposed to composite resin eluates underwent a metabolic activity reduction in a time-dependent manner. After 24 h, a slight metabolic activity decrease could be appreciated for all composite resins. After 48 h and 72 h, a more evident reduction in the metabolic activity for GR and FU was detected, while for BF, the decrease was less pronounced. After a 1-week exposure, a significant metabolic activity decrease was detected in all composite eluates compared to the CTRL group.

In the present study, with the aim of adequately predicting the real biological effects of dental composites, great attention was paid to cell inflammatory and apoptosis patterns and their biological mechanisms. Concerning the inflammatory pathway NFκB p65/MyD88/NALP3, immunofluorescences analyses showed some protein upregulation after a 1-week exposure. Specifically, this pathway activation was significantly higher after GR exposure compared to BF and FU exposure. A more pronounced pro-inflammatory effect should be carefully considered when selecting the appropriate composite in daily clinical practice. According to previous research [48], GR showed a high TEGDMA release after a 24 h soaking in the culture medium. These data would support the results of the present study.

Regarding the apoptosis pathway BCL-2/BAX/CASP-3, a reduction in the BCL-2 immunofluorescence levels was detected in hGFs exposed to dental composites for 24 h and 1 week. hGFs exposed to GR showed a more pronounced BCL-2-expression decrease compared to the control group and to the BF- and FU-exposed cells. However, BAX and CASP-3 levels increased after exposure to all composite eluates (GR, BF, and FU).

Real-Time PCR analysis results regarding *RELA*, *MYD88*, *NLRP3*, *BCL2*, *BAX,* and *CASP3* expression supported the findings achieved by Confocal Microscopy. Specifically, GR eluate exposure led to an upregulation in the inflammatory genes *RELA*, *MYD88*, and *NLRP3* compared to the control group and to the BF and FU eluates. Pro-apoptotic *BAX* and *CASP3* genes expression evidenced a significant upregulation in hGFs exposed for 1 week to GR, while the anti-apoptotic *BCL2* gene expression was downregulated after 1 week of GR exposure compared to the other groups.

The findings herein observed concerning inflammation and apoptosis pathways show time-dependent results, with a stronger effect after a 1-week than a 24 h exposure to the eluates. The pathway activation seemed also material-dependent, being less evident with BF and FU, which suggests a significant role of the composite formulation in the ultimate biological effects.

The composites selected for the present study have a different chemical formulation (Table 1). Based on the information retrieved from the respective manufacturers, GR is the only one of the tested composites that includes Bis-GMA within its organic matrix. This would suggest that it is one of the potential monomers responsible for the more pronounced cytotoxic effects herein observed with GR. Several studies have already demonstrated the pro-inflammatory potential of Bis-GMA [14,15,16], which is mainly related to the presence of Bisphenol A (BPA) derivative in its structure. BPA is a synthetic organic compound that may promote pro-inflammatory cytokines production [49]. Its implication in different diseases has been demonstrated, and its effects seem to be involved in a large variety of pathogeneses of different disorders [50]. However, as already discussed elsewhere [48], it is worth observing that the overall cytotoxicity of a material should be better described as the effect of a complex and synergistic interaction of all its different components, rather than focusing just on one of them.

This in vitro study highlighted specific pathways behind the potential cytotoxic effect of dental composite eluates towards hGFs. From a clinical point of view, a composite material able to minimize or even eliminate the activation of these pathways should be preferred by dental practitioners. According to Schätzle et al. [35], composite restorations placed below the gingival margin can lead to a loss of attachment resulting from inflammation detected 1 to 3 years after the restorative treatment. Conversely, Bertoldi et al. [51] demonstrated that composite restorations were biocompatible with the periodontium; however, they considered just a short-term observation of a 3-month follow-up. The surface polishing of the restoration seems able to affect the cell–biomaterial interface, involving cell attachment, cell proliferation, and the subsequent healing of periodontal tissues [52]. The results of the present study suggest that differences in the composite formulation may also significantly affect the extent of the inflammatory response. As a consequence, resin composites that over-stimulate any adverse or allergic reaction should be considered harmful [32,33,34] for the periodontium. If placed in contact with or close to the periodontal tissues, they may exacerbate the risk for gingival inflammation or even gingival recession and loss of attachment [35]. The choice of the most biocompatible restorative material is therefore of paramount relevance. From this point of view, within the limits of the present investigation, BF and FU seemed to be the least cytotoxic among the resin composites tested herein. However, it should be underlined that not only GR but all the resins tested led to some mediator alteration; thus, to some extent, they could all potentially trigger inflammatory processes.

Clinically, despite not directly investigated in the present research, it could be speculated that the release of detrimental monomers through the dentinal tubules might also determine undesirable pulp reactions, from reversible to irreversible pulpitis and pulp necrosis, eventually compromising the pulp vitality and the long term prognosis of the entire tooth [36].

From the perspective of the dental industry, a better understanding of the different pathways involved in composite cytotoxicity would allow us to formulate sound hypotheses about possible modifications of the present composite formulations (i.e., by including potentially beneficial additives), with the aim of limiting any detrimental pattern activation. Many molecules and compounds have been examined so far in the literature that would seem able to inhibit or antagonize NFκB p65/MyD88/NALP3 and BCL-2/BAX/CASP-3 pathways. For example, regarding the inflammatory pathway, it was found that resveratrol, a natural non-flavonoid polyphenolic of red grape skin [53], is an activator of Sirt1 [54], a class III histone deacetylase that acts on various substrates as NFκB and p53 [55,56], regulating the NALP3 inflammasome [54]. MCC950 is a specific inhibitor blocking the nucleotide-binding domain activation and leucine-rich repeat pyrin domain containing NALP3 inflammasome [57]. Also, other compounds such as BMS-345541 and KPT-8602 can directly inhibit NFκB signaling, reducing NALP3 expression [58]. Regarding the apoptosis pathway, it has been shown that cilostazol, an antiplatelet agent and aphosphodiesterase type-3 selective inhibitor [59], is able to protect against amyloid-β-induced apoptosis by suppressing BAX and upregulating BCL-2 expression [59]. Survivin, an endogenous anti-apoptotic protein, was demonstrated to inhibit CASP-3 activation through XIAP interaction promoting its proliferation [60]. Taghizadeh et al. [61] demonstrated the potential of gliclazide (a second-generation sulfonylurea hypoglycemic drug employed in the treatment of patients with insulin-dependent diabetes mellitus) to reduce the oxidative stress and both CASP-3 and NFκB activities.

On the above bases, as a perspective for subsequent research, further studies seem to be required, in order to better understand whether the inclusion of any of the above-mentioned and potentially beneficial additives into the present commercial composite formulations would lead to a significant reduction in the activation of the inflammation and apoptosis pathway, while not adversely affecting the ultimate mechanical and optical properties of the material.

Many studies have so far focused on residual monomers and the cytotoxicity of dental polymers [62]. The present study differs from the previous ones because it not only investigated the metabolic activity modifications occurring during dental resin exposure but also characterized cell inflammation and apoptosis patterns and their biological mechanism by analyzing cell proliferation, apoptosis, and inflammation-related genes. However, it is worth noting that these short-term in vitro study results should not be straightly used to extrapolate the actual clinical conditions. In the oral cavity, saliva continuously washes away the compounds released by dental polymers, leading to a significant wash-out effect [63], and oral mucosa is definitely more tolerant to harmful compounds than cell cultures. Future research should further examinate the biological effects herein observed, with the aim of expanding the present investigation by including the analysis of other molecular mechanisms that are potentially implicated in inflammation, apoptosis, and necrosis processes.

## 5. Conclusions

In accordance with the present results and within the limitations of an in vitro study, it can be concluded that the inflammatory pathway NFκB p65/MyD88/NALP3 was activated after the exposure to resin composite eluates, with marked metabolic activity reduction after 24 h and 1-week exposure. The inflammation and apoptotic pathway activation were time dependent. Moreover, the cytotoxic effects were also material dependent, which was less evident with BF and FU. This suggests a significant role of the specific material composition in the ultimate biological effects on hGFs culture and provides theoretical foundations for optimizing the industrial processes behind material manufacturing and for a more informed material selection by clinicians.

## Figures and Tables

**Table 1:** Composite resins selected for the study design.

Experimental Group	Material	Manufacturer	Batch	Composition
GR	GrandioSO -Shade A2- (Nanohybrid)	Voco GmbH (Anton-Flettner-Straße 1-3, 27472, Cuxhaven, Germany)	2213772	89% (*w*/*w*) fillers (1 μm glass ceramic filler, 20 nm–40 nm silicon dioxide fillers), Bis-GMA, Bis-EMA, TEGDMA.
BF	Enamel Plus HRi Biofunction -Shade BF2- (Nanohybrid)	Micerium S.p.A (Via Guglielmo Marconi, 83, 16036, Avegno, Genova, Italy)	2024006140	74% in weight (60% in volume) fillers (0.005 μm–0.05 μm silicon dioxide fillers), (0.2–3.0 μm glass fillers), Urethane dimethacrylate, Tricyclodecane dimethanol dimethacrylate.
FU	3M™ Filtek™ Universal Restorative—Shade A2	3M ESPE (2510 Conway Avenue St. Paul, MN 55144-1000 USA)	10544071	Silane treated ceramic (40–70 by wt%) *, silica (1–5 wt%) *, and zirconia (1–5 wt%) *, Ytterbium Fluoride (1–10 wt%) *, Aromatic Urethane Dimethacrylate, Diurethane Dimethacrylate, 1,12-Dodecane Dimethycrylate

* Exact filler content percentage has been withheld by the manufacturer as a trade secret.

**Table 2:** Primer sequences used for real-time PCR reactions.

Gene	Primer 1	Primer 2
	Sequence (5′-3′)	Sequence (5′-3′)
*RELA*	5′-CGAGCTTGTAGGAAAGGACTG-3′	5′-TGACTGATAGC-CTGCTCCAG-3′
*MYD88*	5′-CGGTCTCCTCCACATCCT-3′	5′-GCCGGATCTCCAAGTACTCA’-3′
*NLRP3*	5′-GAATGCCTTGG GAGACTCAG-3′	5′-AGATTCTGATT AGTGCTGAGTACC-3′
*BCL2*	5′-GATGACTGAGTACCTGAACCG 3′	5′-ACCAATCTTGT AGGACTGACC-3′
*BAX*	5′-TCTGAGCAGATCATGAAGACAG-3′	5′-AGCCAGGAGAAATCAAACAGAG-3′
*CASP3*	5′-CTCTGGAATATCCCTGGACAAC-3′	5′GTTTGCTGCATCGACATCTG-3′
*B2M*	5′-GGACTGGTCTT-TCTATCTCTTGT-3′	5′-ACCTCCATGATGCTGCTTAC-3′

## Data Availability

Data is contained within the article.
